# Targeting the *ERG* oncogene with splice-switching oligonucleotides as a novel therapeutic strategy in prostate cancer

**DOI:** 10.1038/s41416-020-0951-2

**Published:** 2020-06-25

**Authors:** Ling Li, Lisa Hobson, Laura Perry, Bethany Clark, Susan Heavey, Aiman Haider, Ashwin Sridhar, Greg Shaw, John Kelly, Alex Freeman, Ian Wilson, Hayley Whitaker, Elmar Nurmemmedov, Sebastian Oltean, Sean Porazinski, Michael Ladomery

**Affiliations:** 1 Institute of Biomedical & Clinical Sciences, University of Exeter Medical School, Exeter, UK; 2 Faculty of Health and Applied Sciences, University of the West of England, Bristol, UK; 3 Molecular Diagnostics and Therapeutics Group, University College London, London, UK; 4 Department of Pathology, UCLH NHS Foundation Trust, London, UK; 5 Department of Urology, UCLH NHS Foundation Trust, London, UK; 6 John Wayne Cancer Institute, Providence Saint John’s Health Center, Santa Monica, USA; 7 Present Address: Faculty of Medicine, St Vincent’s Clinical School, University of NSW, Darlinghurst, Sydney, NSW 2010 Australia

**Keywords:** Prostate cancer, Antisense oligonucleotide therapy

## Abstract

**Background:**

The *ERG* oncogene, a member of the ETS family of transcription factor encoding genes, is a genetic driver of prostate cancer. It is activated through a fusion with the androgen-responsive *TMPRSS2* promoter in 50% of cases. There is therefore significant interest in developing novel therapeutic agents that target *ERG*. We have taken an antisense approach and designed morpholino-based oligonucleotides that target *ERG* by inducing skipping of its constitutive exon 4.

**Methods:**

We designed antisense morpholino oligonucleotides (splice-switching oligonucleotides, SSOs) that target both the 5′ and 3′ splice sites of ERG’s exon 4. We tested their efficacy in terms of inducing exon 4 skipping in two ERG-positive cell lines, VCaP prostate cancer cells and MG63 osteosarcoma cells. We measured their effect on cell proliferation, migration and apoptosis. We also tested their effect on xenograft tumour growth in mice and on ERG protein expression in a human prostate cancer radical prostatectomy sample ex vivo.

**Results:**

In VCaP cells, both SSOs were effective at inducing exon 4 skipping, which resulted in a reduction of overall ERG protein levels up to 96 h following a single transfection. SSO-induced ERG reduction decreased cell proliferation, cell migration and significantly increased apoptosis. We observed a concomitant reduction in protein levels for cyclin D1, c-Myc and the Wnt signalling pathway member β-catenin as well as a marker of activated Wnt signalling, p-LRP6. We tested the 3′ splice site SSO in MG63 xenografts in mice and observed a reduction in tumour growth. We also demonstrated that the 3′ splice site SSO caused a reduction in ERG expression in a patient-derived prostate tumour tissue cultured ex vivo.

**Conclusions:**

We have successfully designed and tested morpholino-based SSOs that cause a marked reduction in ERG expression, resulting in decreased cell proliferation, a reduced migratory phenotype and increased apoptosis. Our initial tests on mouse xenografts and a human prostate cancer radical prostatectomy specimen indicate that SSOs can be effective for oncogene targeting in vivo. As such, this study encourages further in vivo therapeutic studies using SSOs targeting the *ERG* oncogene.

## Background

The human oncogene *ETS*-*related gene* (*ERG*), located on chromosome 21, encodes an E-26 transformation-specific (ETS) family of DNA-binding transcription factors. There are at least 28 ETS family members in humans, involved in a wide range of developmental processes.^1^ All members of the family share a conserved 85 amino-acid region known as the ETS DNA-binding domain that recognises a target sequence containing a core GGA(A/T) motif.^2^ The ETS member *ERG* was first described in 1987 by Rao et al.^3^ The *ERG* oncogene is now considered an oncogenic driver in prostate cancer (PCa).^4,5^ *ERG* is over-expressed in a remarkable 50% of PCa cases because of a 3 Mb deletion that fuses the androgen-responsive *TMPRSS2* promoter with *ERG*.^6^ The fusion most often occurs between *TMPRSS2* exons 1 or 2 and exon 4 of *ERG*.^4^ We have recently shown that ERG expression is increased in patients with advanced PCa and that higher levels of ERG are associated with seminal vesicle invasion (stage T3b) and biochemical recurrence^7^ (prostate-specific antigen (PSA)–only recurrence).

Recent literature has continued to underline ERG’s important role in PCa. The list of oncologically significant transcriptional targets of ERG is growing. These now include the Wnt receptor Frizzled 8 (*FZD8*),^8^ the tumour suppressor phosphatase and tensin homologue (*PTEN*),^9^ and the α1 and β1 subunits of soluble guanylyl cycles (*GUCY1A3* and *GUCY1B3*),^10^ the latter indicating that *TMPRSS2*:*ERG* can activate NO-cGMP signalling in PCa. *TMPRSS2*:*ERG* also enhances the osteoblastic phenotype of PCa bone metastases^11^ and promotes the recruitment of regulatory T cells to promote tumour growth.^12^ In addition to its increasing prominence in PCa, it is also clear that ERG exerts its oncogenic effects in other contexts. For example, *ERG* has been described as a megakaryocytic oncogene that promotes the rapid onset of leukaemia in mice.^13^ Additional complexity in *ERG*’s oncogenic properties arises from alternative splicing. The majority of human multi-exon genes are alternatively spliced including *ERG*. Of particular interest is *ERG*’s cassette exon 7b. Exon 7b is 72 bp long and adds, in-frame, 24 amino-acids to the transcriptional transactivation domain. We have shown that increased exon 7b inclusion is associated with advanced PCa.^7^ The 24 amino-acids encoded by the evolutionarily-conserved exon 7b include an extracellular-signal-regulated kinase (ERK) docking site (with consensus sequence FxFP).^14^ The functional importance of the ERK docking motif encoded by exon 7b is now clear. The phosphorylation of ERG by ERK leads to the dissociation of Polycomb repressive complex 2 (PRC2) resulting in ERG target gene activation. Mutation of the ERK docking site to AAAP prevents phosphorylation of ERG by ERK.^15^ In our hands, and consistent with these findings, the reduction of exon 7b inclusion induced with splice-switching oligonucleotides (SSOs) resulted in decreased cell proliferation, migration and increased apoptosis^14^ confirming that exon 7b contributes to ERG’s oncogenic properties. These findings are consistent with earlier observations by Wang and colleagues who demonstrated that ERG isoforms that include 7b are more efficient in driving cell proliferation.^16^

Given the growing importance of ERG in PCa there is widespread interest in developing ERG-based therapies. One approach is to target co-factors of ERG such as poly ADP-ribose polymerase (PARP) and histone deacetylase (HDAC). Treatment with the PARP inhibitor olaparib reduces the invasive properties of ERG-positive cells^17^ and another PARP inhibitor, rucaparib, sensitises cells to low-dose radiation.^18^ Treatment of ERG-positive cells with the HDAC inhibitors trichostatin A and MS-275 reduces cell growth and increases cell death.^19^ Another approach is to target the ERG transcription factor directly.^20^ The small molecule inhibitor YK-4-279 binds to ERG, interferes with its protein-protein interactions inhibiting its transcriptional activity,^21^ and impedes the growth of patient-derived PCa xenografts in mice.^22^ Cancer-associated genes can also be targeted with antisense oligonucleotides (ASOs).^23,24^ The pharmacodynamic properties of ASOs are improving significantly and there is a wide choice of chemistries available.^25^ Among these, morpholinos are highly stable DNA analogues, which provide excellent results in vivo^26^ as illustrated by the FDA-approved morpholino drug Eteplirsen (Exondys 51, Sarepta Therapeutics Inc.), designed for the treatment of the effects of mutations that cause Duchenne’s muscular dystrophy (DMD), a previously intractable genetic degenerative muscle disease. Eteplirsen functions by causing the skipping of exon 51 of the *DMD* transcript and is proving to be clinically effective.^27^ ASOs can modify target gene expression in several ways.^28^ They can bind proximally to the translation start site and impede translation and in the case of SSOs, they can also interfere with pre-mRNA splicing. SSOs can promote the inclusion of desirable exons (e.g. TOES, targeted oligonucleotide enhancers of splicing,^29^) or induce exon skipping as illustrated by our own ERG exon 7b SSO^14^ and Eteplirsen.^27^ Another success story is Nusinersen, an FDA-approved ASO (Spinraza, Biogen) that is used to treat spinal muscular atrophy (SMA). It alters *survival motor neuron 2* (*SMN2*) pre-mRNA splicing to restore inclusion of a mutated exon 7, increasing the expression of functional SMN protein. Spinraza achieves improved motor milestones and event-free survival in children with this previously intractable genetic condition.^30^

Here we describe the first study to design and evaluate morpholino-based SSOs that target *ERG*’s exon 4. We test their effect on ERG protein expression and cancer cell biology in ERG-expressing cell lines, and their effect on tumour growth in xenografted mice and their ability to disrupt ERG expression ex vivo.

## Methods

### Cell culture

MG63 (ECACC, human osteoblast-like osteosarcoma cells, catalogue no. 86051601) and VCaP cells (ECACC, human prostate cancer vertebral metastasis, catalogue no. 06020201) were grown in DMEM (Gibco, UK) supplemented with 10% (v/v) foetal bovine serum (Sigma-Aldrich, UK) and 2 mM glutamine at 37 °C, in a 5% CO_2_ humidified incubator.

### Vivo morpholinos

All vivo-morpholino SSOs) were purchased from Gene Tools, LLC, USA. SSOs were designed against both the 5′ and 3′ splice-sites of ERG exon 4. The sequence of the ERG exon 4 5′ splice-site-targeting SSO (E45′) was 5′-GGCGGAAGTCTCCTTACCTTGAGCC-3′ and for the ERG exon 4 3′ splice-site (E43′) the SSO sequence was 5′-GCTTCCTGAATGCCCAAAGAAACAC-3′. The sequence for the control SSO in vitro, targeting an intron in a ß-globin gene variant associated with ß-thalassaemia was 5′-CCTCTTACCTCATTACAATTTATA-3′. For in vivo and ex vivo experiments, the sequence for the scrambled control SSO was 5′-GACAATATAGGACGCCACCGCAACC-3′. Stocks of each vivo morpholino were prepared in sterile tissue culture grade H_2_O (Gibco, UK) at a concentration of 0.5 mM. Each SSO had an octa-guanidine dendrimer moiety to facilitate delivery for cellular uptake and was added directly to culture media. For experiments with VCaPs, the transfection reagent endoporter (Gene Tools, LLC, USA) was used at 10 μM to facilitate the uptake of SSOs. The Gene Tools recommended working concentration range for vivo-morpholinos in cell culture is 1–10 μM; we therefore performed experiments within this range.

### RNA extraction, cDNA synthesis and PCR

For in vitro experiments, total RNA was extracted using a total RNA isolation mini kit (Agilent Technologies Ltd) according to manufacturer’s instructions. All samples were treated with DNAse on the columns using RNase-free DNase I provided in the kit. For in vivo and ex vivo experiments, total RNA was extracted using mechanical homogenisation of samples with Qiazol (Qiagen) according to manufacturer instructions. cDNA was synthesised from 0.2 to 1 μg of total RNA using a Precision nanoScript 2 Reverse transcription kit (Primerdesign Ltd, UK), according to manufacturer instructions.

Hot Start Taq 2X master mix (New England Biolabs (NEB), UK) was used for standard PCR according to manufacturer instructions. Primers for detecting ERG exon 4 skipping were as follows, forward: 5′-TTTGGAGACCCGAGGAAAGC-3′, reverse: 5′-AGAGAAGGATGTCGGCGTTG-3′. The final concentration for each primer in the reaction was 0.4 μM. PCRs were run as follows: initial denaturation at 95 °C for 30 s, then 30 cycles of 95 °C for 30 s, 54 °C for 1 min, 68 °C for 1 min and a final extension at 68 °C for 5 min.

Gels were imaged on a Li-Cor Odyssey Fc imaging system (Li-Cor Ltd). Splice isoform ratios were determined by measuring the relative brightness of PCR bands compared to each other using gel Image Studio Lite software (Li-Cor Ltd). Percent spliced in (PSI) was determined as a ratio of the intensity of the top band (exon included) to the total signal of both bands. Following imaging, PCR bands were excised using a Monarch DNA Gel Extraction Kit (NEB) according to manufacturer’s instructions and exon skipping was confirmed by sequencing with Eurofins Scientific (Germany). Sequences from Eurofins were aligned with ERG exon 4 reference sequences from NCBI (NIH) using ApE plasmid editor v2.

### Western blotting

Lysates were prepared using RIPA buffer (10 mM Tris-Cl (pH 8.0), 1 mM EDTA, 1% (v/v) Triton X-100, 0.1% (w/v) sodium deoxycholate, 0.1% (w/v) SDS and 140 mM NaCl) supplemented with protease inhibitor tablets (ThermoFisher, UK). Equal protein samples were separated on 10% (v/v) SDS polyacrylamide gel electrophoresis gels and transferred to PVDF membranes (Sigma Aldrich) which were blocked and probed overnight at 4 °C with 1:1000 anti-ERG (Abcam, UK), 1:10,000 anti-β-actin (Abcam), 1:2500 anti-GAPDH (Millipore, UK), 1:1000 β-catenin, 1:1000 anti-c-Myc, 1:1000 anti-cyclin D1 or 1:1000 anti-phospho-LRP6 (all Cell Signalling Technologies, UK) primary antibodies. Membranes were incubated in 1:2000 HRP-linked anti-rabbit or anti-mouse IgG secondary antibody (NEB) for 2 h at room temperature. Membranes were incubated in Luminata Forte Western HRP substrate (Millipore) for chemiluminescent detection prior to image acquisition using a Li-Cor Odyssey imaging system.

### Analysis of cell proliferation and apoptosis

For proliferation and apoptosis assays cells were seeded on coverslips in 6-well plates and treated with SSOs for 48–96 h. To analyse proliferation, SSO-treated cells were fixed in 4% (v/v) PFA for 15 min and permeabilised with 0.1% (v/v) Triton X-100 followed by blocking in 3% (v/v) FBS for 1 h. Cells were incubated with rabbit Ki67 primary antibody (Abcam) in blocking solution (1:200) for 2 h at room temperature followed by incubation for 1 h in 1:200 AlexaFluor 568 (ThermoFisher). For apoptosis assays, 45 min prior to the end of the SSO incubation period, CellEvent Caspase-3/7 reagent was added according to manufacturer instructions. For both assays, cells were counterstained in 2 μg/ml Hoechst 3342, mounted in mowiol (Sigma Aldrich) and representative images were taken using an Eclipse 80i microscope (Nikon). ImageJ (v2.0-rc-69/1.52i) software was used to calculate the percentage of Ki67+ and Caspase-3/7+ cells.

### CD31 immunofluorescence

Frozen sections from xenograft tumours were cut as 7 µm thick sections and fixed in 4% paraformaldehyde solution in PBS (ThermoFisher) for 10 min at room temperature. Fixed tumour tissues were washed in TBS and permeabilised with TBS with Triton X-100 (TBS/T, 0.1% v/v) for 30 min at room temperature. Tissues were blocked with 10% normal goat serum (NGS) in 1% BSA in TBS/T for 1 h at room temperature before incubating with rabbit anti-CD31 antibody (Abcam) diluted 1:50 in 1% BSA in TBS/T, overnight at 4 °C. Sections were washed in TBS/T and incubated with Alexafluor 568 goat anti-rabbit antibody (ThermoFisher) at 1:750 dilution in 1% BSA in TBS/T for one hour at room temperature. Sections were washed with TBS and mounted with mounting medium with DAPI (Abcam). Images were taken at 20x magnification with Leica DM4000 B LED Fluorescence microscope.

### Cell migration assays

For migration assays, PET inserts (8 μm membrane pore size; Millipore) were placed in 24-well plates and 1 × 10^5^ of 24 h SSO-treated cells were added to the upper chamber of the insert in serum free media. In all, 600 μl of medium with 10% (v/v) FBS was added to the lower chamber of the 24-well plate and cells were incubated for 48 h. Cells remaining on the upper membrane of the insert were removed and inserts were fixed in 4% (v/v) PFA followed by staining with Hoechst for quantifying migrated VCaP cells. The inserts were imaged with an Eclipse 80i microscope and the number of cells present on the lower membrane of the inserts was quantified with ImageJ. For quantifying migrated MG63 cells, inserts were PFA fixed and stained using 0.2% (v/v) crystal violet in 2% (v/v) methanol. Inserts were then placed into 0.1% (v/v) SDS in PBS and the crystal violet residue in the SDS-PBS was quantified by transferring to a 96-well plate and measuring absorbance at 590 nm.

### TopFlash luciferase reporter assays

MG63 and VCaP cells were seeded in six-well plates at a density of 500,000 cells per well in 1.5 ml of medium. Cells were transfected with a cocktail of the following plasmids: 1 µg TopFlash and 50 ng pRL-TK using Lipofectamine 2000 (Invitrogen). After 24 h, cells were trypsinised and seeded in 96-well plates at a density of 10,000 cells per well in 100 µM of medium. After 24 h, SSOs were diluted in media supplemented with endoporter (Gene Tools), then added to the cells with final concentrations of 0.1, 0.3, 1.0, 3.0 and 10.0 µM, in triplicates. Following 24 h of incubation, luciferase reporter activity was measured using the Dual-Glo system (Promega) on a plate reader. Relative luciferase activity was normalised to non-SSO controls.

### Mouse xenograft analysis

Two-month-old male nude mice (CD1; Charles River, USA) were housed under pathogen-free conditions. All animal operations were approved by the Animal Ethics Committee, University of Exeter, U.K. For heterotopic xenografts, 7 × 10^6^ MG63 cells resuspended in 100 μl of PBS were injected subcutaneously into the right flank of mice. Tumours were measured by calliper twice weekly and tumour volume was calculated according to the formula: [(length + width)/2] × length × width. Once tumours reached 3 mm × 3 mm in size, mice were randomly assigned to treatment groups so that combined tumour volumes were equivalent between groups. 12.5 mg/kg of either SSO (in PBS) or PBS alone was administered by intraperitoneal (I.P.) injection twice weekly, for each mouse in the morning in home cages, for the duration of the study (56 days). Systemic delivery was chosen since it more accurately reflects delivery methods likely to be applied in the clinic. Following the study, mice were euthanised by schedule 1 cervical dislocation and tumours were harvested for RNA and protein extraction.

### Ex vivo analysis

Tissue sampling from radical prostatectomy specimens was performed using the PEOPLE methodology^31^ and ex vivo culture of tissue was performed as previously described.^32^ Briefly, gelatine sponges were placed in 24-well plates with 200 μl of RPMI supplemented with 10% (v/v) FBS 2-3 h prior to tissue harvest to allow the sponges to draw up the media. For treatment groups, 10 μM SSO was added to the RPMI. Fresh 6 mm cores were divided using a scalpel and placed on the sponges, then incubated for up to 72 h followed by harvesting for histology and RNA/protein extraction as described above. For histological analysis, samples were fixed in 10% (v/v) neutral buffered formalin and stored as FFPE blocks. In all, 4 μm sections from the FFPE blocks were stained with haematoxylin and eosin (H&E) to assess tumour content of samples as per the 100,000 Genomes Project standard operating procedures (https://www.genomicsengland.co.uk/about-genomics-england/the-100000-genomes-project/information-for-gmc-staff/sample-handling-guidance). Slides were assessed by an experienced consultant uropathologist and tumour content was reported as 0–100% in 5% increments. For ERG immunohistochemistry, 4 μm sections from the FFPE blocks were stained on the BondMax autostainer (Leica) with anti-ERG antibody (Abcam, ab92513; 1:200 dilution).

### Statistical analysis

ANOVAs (with Dunnet’s tests where appropriate) or *T*-tests were carried out using GraphPad Prism 8 software. Significance levels are indicated by asterisks where * = *P* < 0.05, ** = *P* < 0.01, and *** = *P* < 0.001 and are based on comparisons with untreated controls unless stated otherwise. Data are reported as means and error bars show 95% confidence intervals of means.

## Results

SSOs targeting *ERG* cause exon 4 skipping and a reduction in ERG protein levels in ERG-positive cancer cells

**Fig. 1:**
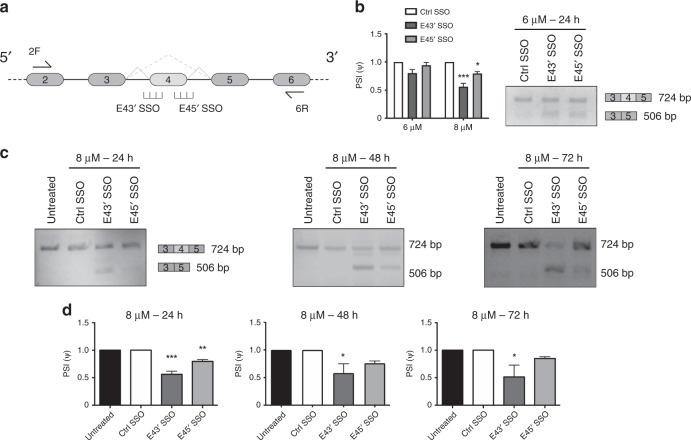
Validation of splice-switching oligonucleotides targeting *ERG* exon 4 in VCaP cells. **a** Schematic showing targeting of *ERG* exon 4 (E4) with splice-switching oligonucleotides (SSOs) designed against the 3′ (E43′) or 5′ (E45′) splice sites (see Supplementary Fig. 1a for additional SSO sequence details). Skipping of exon 4 is denoted by the dashed line. PCR primers to detect the presence or absence of exon 4 are indicated (2F and 6R). **b** Quantification of dose-dependent exon 4 skipping in VCaP cells treated with E4 SSOs for 24 h (*n* = 3, except for ctrl SSO at 6 µM, *n* = 2). A representative RT-PCR of exon 4 skipping is shown on the right. **c** Representative RT-PCR panels showing exon 4 skipping after 24–72 h treatment of VCaP cells with 8 µM E4 SSOs. **d** Quantifications of exon 4 skipping in VCaP cells treated with 8 µM E4 SSOs for 24–72 h (*n* = 3 at all timepoints). *** = *p* < 0.001, ** = *p* < 0.01, * = *p* < 0.05. Ctrl SSO control SSO.

To investigate whether we could disrupt pre-mRNA splicing of the *ERG* oncogene in the context of prostate cancer (PCa), we first designed SSOs targeting the 3′ (E43′) and 5′ (E45′) splice-sites of exon 4 of *ERG* (Fig. 1a, Supplementary Fig. 1a). Exon 4 is 218 bp in size, and its skipping results in a frame-shift and premature termination codon (PTC) leading to nonsense-mediated decay (NMD) of the transcript. Specifically, if exon 4 is skipped, the frameshift produces a stop codon nine bases into exon 5, so that only 20 amino-acids of ERG are translated (MIQTVPDPAAHIKEALS*ELS*stop, where the amino-acids *ELS* are provided by a frameshifted exon 5). Therefore, even if a proportion of exon 4-skipped mRNA escapes NMD and is translated, no viable ERG protein is produced. We treated the VCaP PCa cell line with a single dose of either the E43′ or E45′ SSOs and observed that both SSOs could cause skipping of exon 4 in *ERG* mRNA as assessed by reverse-transcription PCR (RT-PCR) from as early as 24 h up to 72 h following dosing (Fig. 1b, c). Additionally, for both SSOs we observed a dose-dependent effect on exon 4 skipping with increasing dose from 6 to 8 μM (Fig. 1b). By quantifying percentage spliced in (PSI) as a measure of exon 4 skipping, the E43′ SSO appeared more efficient at causing exon 4 skipping, as PSI values for the E43′ SSO were consistently lower than the E45′ SSO and significantly lower versus untreated and control SSO-treated cells from 24 to 72 h (Fig. 1d). We confirmed the skipping of exon 4 in both E43′ and E45′ SSO-treated PCR products by sequencing (Supplementary Fig. 1b). Since we observed partial but significant skipping of exon 4 at 8 μM doses and minimal cell toxicity (some toxicity was observed at 10 μM, data not shown), we proceeded with this dose for subsequent cell biology assays.

**Fig. 2:**
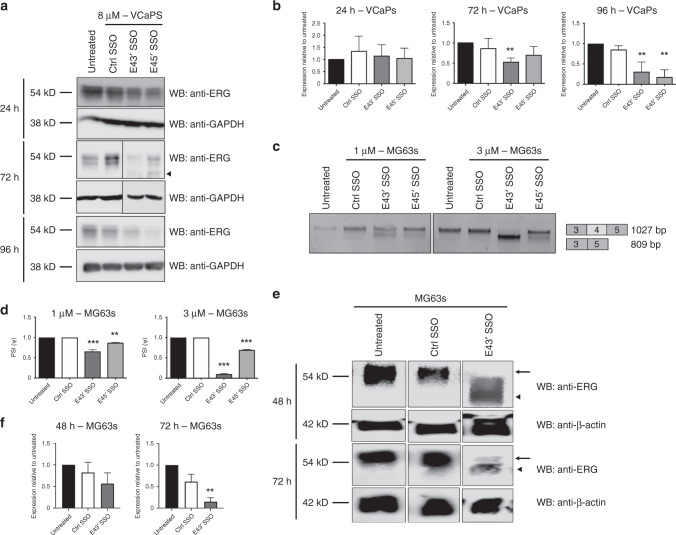
Splice-switching caused by *ERG* exon 4 SSOs reduces ERG protein levels in two cancer cell lines. **a** Representative ERG and GAPDH (loading control) western blots of lysates from VCaP cells treated with 8 µM SSOs for 24, 72 and 96 h. The arrowhead indicates a putative truncated ERG isoform (full length is 54kD). **b** Quantifications of ERG western blots from VCaP cells treated with SSOs for 24–96 h (*n* = 4 for 24 h and 72 h, *n* = 3 for 96 h, except for ctrl SSO at 96 h, *n* = 2). **c** Representative RT-PCR panels for MG63 cells treated with 1 or 3 µM for 24 h. **d** Quantification of exon 4 skipping in MG63 cells treated with SSOs at 1 or 3 µM for 24 h (*n* = 3). **e** Representative ERG and β-actin (loading control) western blots of lysates from MG63 cells treated with SSOs for 48 and 72 h. The arrow indicates full-length ERG isoform (54 kD) and arrowhead indicates truncated ERG isoform. **f** Quantifications of ERG western blots from MG63 cells treated with 5 µM SSOs for 48 and 72 h (*n* = 8 for untreated and ctrl SSO, for E43′ *n* = 4 at 48 h; *n* = 6 at 72 h). ERG protein expression levels were normalised to GAPDH. *** = *p* < 0.001, ** = *p* < 0.01, * = *p* < 0.05. Ctrl SSO control SSO.

We next asked whether these SSO-induced changes in *ERG* at the mRNA level resulted in changes at the protein level. Western blotting demonstrated that at 24 h after E43′ and E45′ SSO treatment, ERG levels were largely unchanged (Fig. 2a, b). However, by 72 h following SSO transfection, VCaP cells displayed clearly reduced ERG protein levels; by 96 h ERG levels were even lower (Fig. 2a, b). As expected, there was a time lag between SSO transfection and NMD of *ERG* mRNA and subsequent depletion of ERG protein; and the effect of a single SSO transfection on ERG expression persisted over several days. We confirmed these findings using another ERG-positive cancer cell line, the MG63 osteosarcoma cell line. We obtained a similar result when treating MG63 cells with single doses of E43′ and E45′ SSOs, with more efficient skipping of exon 4 induced by the E43′ SSO (Fig. 2c, d). We therefore examined ERG protein levels in MG63 cells treated with the more efficient E43′ SSO and observed a significant reduction in ERG level by 72 h (Fig. 2e, f). Taken together, these results suggest that the use of *ERG*-targeting SSOs is a viable approach for reducing ERG protein expression in multiple cancer cell lines.

### SSOs targeting *ERG* affect PCa cell biology via down-regulation of Wnt/β-catenin signalling

**Fig. 3:**
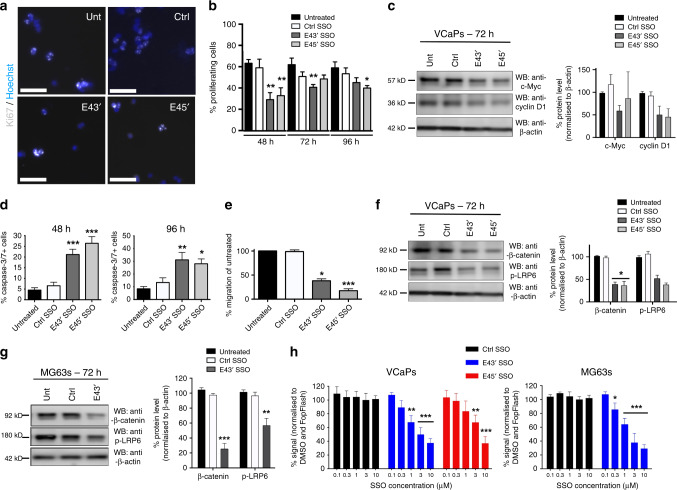
*ERG* exon 4 SSOs affect VCaP cancer cell behaviour and signalling. **a** Representative immunofluorescence images for Ki67 (grey) and Hoechst (blue) after 48 h of 8 µM E4 SSO treatment in VCaP cells. **b** Quantification of Ki67+ VCaP cells after 48–96 h of 8 µM E4 SSO treatment (*n* = 3 for all time points). **c** Western blotting and quantification for regulators of cell cycle progression, cyclin D1 and c-Myc, following 72 h of 8 µM E4 SSO treatment in VCaP cells (*n* = 3). β-actin was used as loading control. **d** Caspase-3/7 staining of VCaP cells treated with E4 SSOs at 8 µM for 48 h and 96 h (*n* = 3 at all timepoints). **e** Quantification of VCaP cells migrated in transwell assays after 48 h of E4 SSO treatment at 8 µM (*n* = 4, except for untreated where *n* = 3). **f** and **g** Representative western blotting for and quantification of key components of the canonical Wnt signalling pathway (β-catenin and p-LRP6) following 72 h of SSO treatment in VCaP cells (8 µM) and MG63 (3 µM) cells respectively (*n* = 3). β-actin was used as loading control. **h** TopFlash assays to assess Wnt pathway activity following 72 h of SSO treatment in VCaP cells and MG63 cells. *** = *p* < 0.001, ** = *p* < 0.01, * = *p* < 0.05. Unt untreated; Ctrl SSO control SSO. Scale bar = 40 µm.

Over-expression of ERG in PCa is associated with disease initiation, progression and spread.^4^ This ERG over-expression leads to downstream signalling that drives PCa cell proliferation, survival, migration and metastasis. We examined several of these cancer cell hallmarks following treatment of VCaP cells with single SSO doses and observed a significant reduction in VCaP cell proliferation in SSO-treated cells 48–96 h following dosing as assessed by Ki67 staining (Fig. 3a, b). In support of this, we found that the cell cycle regulator cyclin D1 appeared downregulated following SSO transfection (Fig. 3c). Interestingly, we observed that expression of c-Myc, a potent regulator of cellular proliferation,^33^ which has been shown to be activated by *TMPRSS2*-*ERG* fusions in PCa^33^ and is associated with disease onset and progression^34,35^ was also reduced in VCaP cells upon treatment with the E43′ SSO (Fig. 3c).

Since we observed significant reductions in the proliferative capacity of SSO-treated VCaP cells, we next analysed whether SSO treatment also affected cell survival. We observed that reduced VCaP proliferation was mirrored by marked increases in levels of apoptosis following treatment with SSOs for 48–96 h (Fig. 3d), as determined by caspase-3/7-positivity. We next we asked whether exon 4 SSOs affected VCaP cell migration. Using transwell chamber assays, we established that E43′ and E45′ SSO treatment significantly reduced the ability of VCaP cells to migrate through transwell membranes (Fig. 3e, Supplementary Fig. 2a).

ERG has been shown to drive the Wnt/β-catenin signalling in the context of PCa.^8,36^ Since we observed that levels of the Wnt targets cyclin D1^37^ and c-Myc^38^ were decreased by SSO treatment, we asked whether our ERG-targeting SSOs had an effect on Wnt/β-catenin signalling. Western blotting of SSO-treated VCaP cells revealed a reduction in Wnt/β-catenin signalling as indicated by a reduction in the level of the active upstream canonical LDL receptor LRP6 (p-LRP6) and the key Wnt transcriptional co-regulator β-catenin (Fig. 3f). We then confirmed that a significant reduction in migratory ability (Supplementary Fig. 2b) and Wnt/β-catenin signalling was also present in MG63 cells treated with the E43′ SSO (Fig. 3g). In addition, we also observed significant reductions in cyclin D1 and c-Myc protein levels in MG63s following treatment with the E43′ SSO (Supplementary Fig. 2c). Finally, we used TopFlash assays to directly measure Wnt/β-catenin signalling activity and observed a significant reduction in pathway activity in VCaP and MG63 cells treated with SSOs (Fig. 3h). Collectively these data suggest that the reduced ERG protein levels caused by SSOs reduce the tumorigenicity of PCa cells in vitro, and that this may be due in part to altered Wnt signalling.

### SSOs targeting *ERG* reduce tumour formation in vivo

**Fig. 4:**
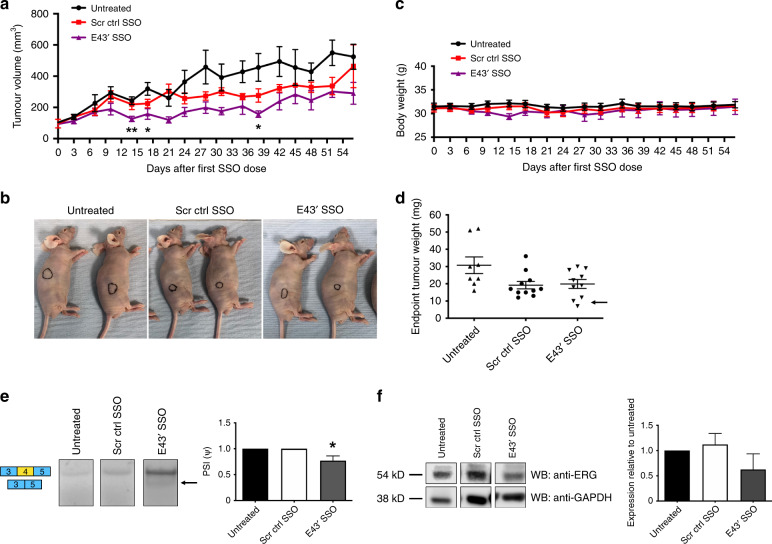
ERG exon 4 SSOs have anti-tumour effects in vivo. **a** Subcutaneous tumour growth of the ERG + MG63 cell line was measured twice weekly following IP injection of E43′ SSOs. Tumour measurements and dosing were performed on the same day. **b** Representative images of subcutaneous tumours (indicated by highlighted regions) in situ. **c** Measurements of body weight following systemic administration of SSOs. **d** Endpoint tumour weights from mice treated with SSOs (*n* = 8 for untreated; *n* = 11 for scrambled ctrl SSO; *n* = 10 for E43′ SSO). **e** Representative RT-PCR panels (left) and quantifications (right) of exon 4 skipping (arrow) from tumour tissue extracted at endpoints (*n* = 5 for untreated; *n* = 7 for scrambled ctrl and E43′ SSOs). **f** Representative panels for ERG and GAPDH (loading control) western blotting (left) in tumour tissue extracted from mice at endpoints. Quantification of ERG western blotting in tumours shown on right (*n* = 3 for untreated; *n* = 7 for scrambled ctrl SSO; *n* = 8 for E43′ SSO). ERG protein expression levels were normalised to GAPDH. *** = *p* < 0.001, ** = *p* < 0.01, * = *p* < 0.05. Scr ctrl SSO Scrambled control SSO.

Since the E43′ SSO demonstrated more efficiency in vitro, and particularly in the context of MG63 cells, we sought to test the ability of this SSO to affect tumour progression in vivo. Using a xenograft model, we subcutaneously injected 7 × 10^6^ of the faster-growing ERG-positive MG63 cells into nude mice and allowed tumours to establish to 3 mm × 3 mm. Mice were then administered with SSOs twice weekly by I.P. injection and tumour volumes were monitored over a period of 56 days. We observed that tumours from mice injected with the E43′ SSO were slower growing versus untreated and control tumours and significantly smaller in volume at several timepoints (Fig. 4a). E43′ SSO-treated tumours appeared smaller in size (Fig. 4b) and had reduced weight versus untreated tumours (Fig. 4d). We also saw a partial effect of the control SSO on tumour growth (Fig. 4a, b, d), suggesting a mild toxic or off-target effect, potentially due to the guanidinium mini-dendrimer present in the vivo-morpholinos; however, overall both SSOs appeared well tolerated by the mice (Fig. 4c), which was as expected, since we confirmed minimal complementarity between the mouse *ERG* gene and our E43′ SSO (Supplementary Fig. 3a). Furthermore, there were several E43′-treated tumours with lower weights than in the control SSO mice (Fig. 4d, arrow) suggesting specific effects of the E43′ SSO on tumour growth. To confirm that this reduced tumour growth was due to effects of the E43′ SSO on ERG splicing and protein levels within the tumour, we harvested RNA and protein from treated tumours. RT-PCR revealed a small but significant increase in skipping of exon 4 in treated tumours (Fig. 4e). Moreover, western blotting indicated reduced levels of ERG protein in E43′ SSO-treated tumours versus untreated and control tumours (Fig. 4f), suggesting incorporation of the SSOs into tumours. We also confirmed that the E43′ SSO had no effects on endogenous endothelial ERG within the tumours by staining tumour sections with CD31 to assess blood vessel density (Supplementary Fig. 3b, c). Taken together, these data suggest the E43′ SSO is stable in vivo when delivered systemically, achieving efficient knockdown of ERG to reduce tumour growth.

### SSOs reduce ERG protein levels in patient-derived prostate cancer samples ex vivo

The ability to target specific areas of tumour and benign tissue within radical prostatectomy specimens to produce high quality material for research purposes is becoming increasingly important for studies seeking to assess the efficacy of new therapeutic strategies. As such we employed the recently published PEOPLE method^31^ to obtain fresh radical prostatectomy specimens for ex vivo culture.^32^ Here we tested the more efficacious E43′ SSO using fresh 6 mm cores from radical prostatectomy samples, which were grown on gelatine sponges (Supplementary Fig. 4a) and treated with SSOs. We obtained multiple cores from the tumours of five patients; however, we could only detect ERG expression in one of the samples designated PPL-0209 (Supplementary Fig. 4b, right panel). For PPL-0209, the patient had a PSA of 14.4 and from their MRI a Likert score of 4 was assigned. Patient age at surgery was 65 years and following a firm digital rectal exam all 9 cores of his transrectal ultrasound biopsy were positive for tumour with a Gleason score of G4 + 3 (Supplementary Fig. 4b, left panel). Clinical stage was T3bN1 and tumour volume was 13.67 ml. Interestingly, for cores obtained from this sample, treatment with the E43′ SSO for 24 h resulted in reduced ERG protein levels as assessed by western blotting when compared to untreated and scrambled control SSO samples (Supplementary Fig. 4c, d). We have previously demonstrated that ERG represses the transcription of the tumour suppressor gene *PTEN* in prostate cancer cells.^9^ In support of this, in parallel to ERG reduction, we noted an increase in PTEN protein expression in the E43′ SSO-treated sample (Supplementary Fig. 4c, e). Although more patient samples and detailed further analyses of the consequences of this ERG reduction for tumour biology are required, this pilot data is an encouraging proof of principle for the use of SSOs to target ERG therapeutically in PCa.

## Discussion

ERG is commonly over-expressed in PCa (~50–55% of cases), most often due to fusions with the androgen-responsive *TMPRSS2* promoter.^6^ ERG is considered a major driver of PCa and is thought to be responsible for many PCa cell traits.^4^ Androgen deprivation therapy (ADT) is a widely used approach to prevent disease progression.^39^ However, nearly all prostate cancers eventually become resistant to ADT,^40^ meaning that there is a clinically unmet need for new therapies targeting the inappropriate activation of *ERG* due to *TMPRSS2* fusions. Here we sought to test a novel approach using antisense morpholinos that cause exon skipping (SSOs) to disrupt pre-mRNA splicing of *ERG* to reduce ERG protein levels and mitigate oncogenic phenotypes driven by ERG.

We first designed SSOs targeting the 3′ and 5′ splice-sites of the constitutive exon 4 of ERG, which we termed E43′ and E45′ respectively. We aimed to induce the skipping of exon 4 causing a frameshift and a resultant introduction of premature termination codons (PTCs) into the reading frame. We hypothesised that this approach would lead to NMD of *ERG* transcripts, thus offering a way to mitigate the over-expression of ERG seen in PCa. We found that both E43′ and E45′ SSOs were able to cause skipping of exon 4 as confirmed by RT-PCR and sequencing of PCR products, which demonstrated the absence of exon 4 sequences in the smaller PCR product obtained from the SSO-treated ERG-positive PCa VCaP cell line, as well as the ERG-positive osteosarcoma MG63 cell line. Exon 4 skipping subsequently led to a reduction in ERG protein levels in VCaP and MG63 cells. ERG is thought to drive proliferation, survival, migration and invasion of PCa cells, and our current study supports this as in the VCaP cell line we observed that decreased ERG levels due to SSO treatment led to reduced cell proliferation, increased cell death and reduced cell migration. It has been established that ERG drives the oncogenic properties of PCa cells through Wnt/β-catenin activation and in line with this we saw reduced activation of the Wnt receptor LRP6 (p-LRP6) and reduced levels of the downstream executor of the pathway β-catenin, alongside reduced expression of Wnt pathway genes cyclin D1 and c-Myc upon SSO transfection. Furthermore, TopFlash assays revealed reduced Wnt/β-catenin pathway activity. Interestingly, we observed differences in the efficacy of exon 4 skipping and resultant ERG reduction induced by the E43′ and E45′ SSOs. It is difficult to predict a priori how SSOs will interact with pre-mRNA structure, so it is possible that the E45′ SSO was less effective due to more limited access to the target sequence during splicing. Alternatively, it may also be due to the E45′ SSO itself forming a secondary structure inhibiting its binding to the 5′ splice-site. Moreover, 5′ splice-site selection by the spliceosome is a complex process affected by many factors and there are many different consensus sequences possible that can act as a 5′ splice-site,^41^ meaning that the steric blocking of the spliceosome at this site may not be as effective as expected. Of note, we have recently reported a similar phenomenon with SSOs targeting exon 7b of ERG, whereby SSOs targeting the 3′ splice-site were also more effective than those targeting the 5′ splice-site.^14^

Here we have also demonstrated the efficacy of targeting *ERG* with SSOs in vivo using xenograft models and ex vivo using patient-derived PCa tumour samples, supporting the approach of targeting an oncogene with SSOs, as others have also previously shown.^42,43^ Mouse xenograft models using the ERG-positive MG63 cell line exhibited reduced tumour growth upon systemic E43′ SSO treatment, accompanied by reduced ERG protein levels in treated tumours harvested from the mice at the study’s conclusion. Importantly, the SSOs appeared well tolerated by the mice following systemic SSO delivery. In future, it will be necessary to ascertain any off-target effects at the molecular level in various organs such as the liver and kidneys where SSOs could become concentrated following intraperitoneal administration, as well as comprehensive analysis of SSO effects on tumour biology in vivo, and whether or not Wnt signalling is also disrupted in this context. Although the scrambled control SSO exhibited mildly-toxic or off-target effects on tumour growth, new less toxic in vivo delivery systems are currently being developed and should help to mitigate this in future studies. In a pilot ex vivo experiment we observed a substantial reduction in ERG protein level and a concomitant increase in PTEN protein level in a primary PCa patient sample treated with the E43′ SSO. These results will need to be supplemented by further studies on larger numbers of PCa patient samples as well as detailed analysis of the effects of ERG knockdown on the cell biology and signalling within patient-derived tumours. Finally, as well as providing a new potential therapeutic approach for the treatment of PCa patients, since *ERG* rearrangements and fusions are also present in Ewing’s sarcoma^44^ and in acute myeloid leukaemia,^45^ and no doubt in other neoplasms, the use of SSOs to target *ERG* may be a suitable therapeutic approach in several cancer types.

## Supplementary information

Supplementary material

## Data Availability

Materials, data and associated protocols are available upon request. Supplementary information is available for this paper online.
