# Supplementary material for: Targeting the ERG oncogene with splice-switching oligonucleotides as a novel therapeutic strategy in prostate cancer
Source: Br J Cancer. 2020 Jun 25;123(6):1024–32. doi: 10.1038/s41416-020-0951-2 (PMC7493922; doi:10.1038/s41416-020-0951-2)
Supplement: Supplementary file 1 — Supplementary material [file 41416_2020_951_MOESM1_ESM.pdf]

Supplementary Fig. 1 SSO-induced ERG exon 4 skipping is confirmed by sequencing of PCR bands. a Schematic showing sequences of *ERG* mRNA targeted by E43′ and E45′ SSOs. b RT-PCR products from SSO-treated VCaP cells were sequenced and aligned with the *ERG* exon 4 sequence (from NM_182918.3) to assess the presence or absence of exon 4 sequence in the PCR product. Red areas show regions of sequenced PCR products outside the sequence of exon 4.

Supplementary Fig. 2 ERG exon 4 SSOs affect VCaP and MG63 cancer cell behaviour. a Representative Hoechst images of VCaP cells migrated in transwell assays after 48 h of E4 SSO treatment at 8 μM. b Quantification of MG63 cells migrated in transwell assays after 48 h of E43′ SSO treatment at 3 μM by crystal violet residue absorbance (n = 3 for all groups). c Western blotting and quantification for regulators of cell cycle progression, cyclin D1 and c-Myc, following 72 h of 3 μM E43′ SSO treatment in MG63 cells. β-actin was used as loading control.*** = $p<0.001$, ** = $p<0.01$, * = $p<0.05$. Ctrl SSO: control SSO. Scale bar = 100 μm.

Supplementary Fig. 3 ERG exon 4 SSOs do not affect endogenous *ERG* in vivo. a Alignment of exon 4 SSOs with mouse ERG. Note that the antisense sequence of the SSOs is shown in the alignments. Alignment shows very poor conservation between mouse and human, especially at the 3′ SSO target site, making cross-reactivity highly unlikely. b Representative panels for CD31 (grey) and DAPI (blue) immunofluorescence on frozen sections of E43′ SSO-treated MG63 subcutaneous tumours to assess tumour blood vessel density. c Quantification of CD31+ area per field of view in representative immunofluorescence images (n = 3 tumours per group, 2 sections per tumour analysed). Scale bars: 75 μm. Scr ctrl SSO: Scrambled control SSO.

Supplementary Fig. 4 ERG exon 4 SSOs demonstrate efficacy in human prostate tumour samples cultured ex vivo. a Schematic showing ex vivo culture system for human radical prostatectomy tumour samples. b Left panel shows hematoxylin and eosin staining of an ERG+ tumour that was cultured ex vivo with a Gleason score of G4+3. Right panel shows representative ERG immunohistochemistry on a tumour section from patient PPL-0209. c ERG, PTEN and GAPDH (loading control) western blotting of lysates from prostate tumour samples cultured ex vivo. d Quantification of ERG protein levels in tumours treated with 10 μM E43′ SSO ex vivo (n = 1 for all). e Quantification of PTEN protein levels in tumours treated with 10 μM E43′ SSO ex vivo (n = 1 for all). For d and e, ERG and PTEN protein expression levels were normalised to GAPDH. Scale bars: left panel = 100 μm, right panel = 250 μm. Scr ctrl SSO: Scrambled control SSO.

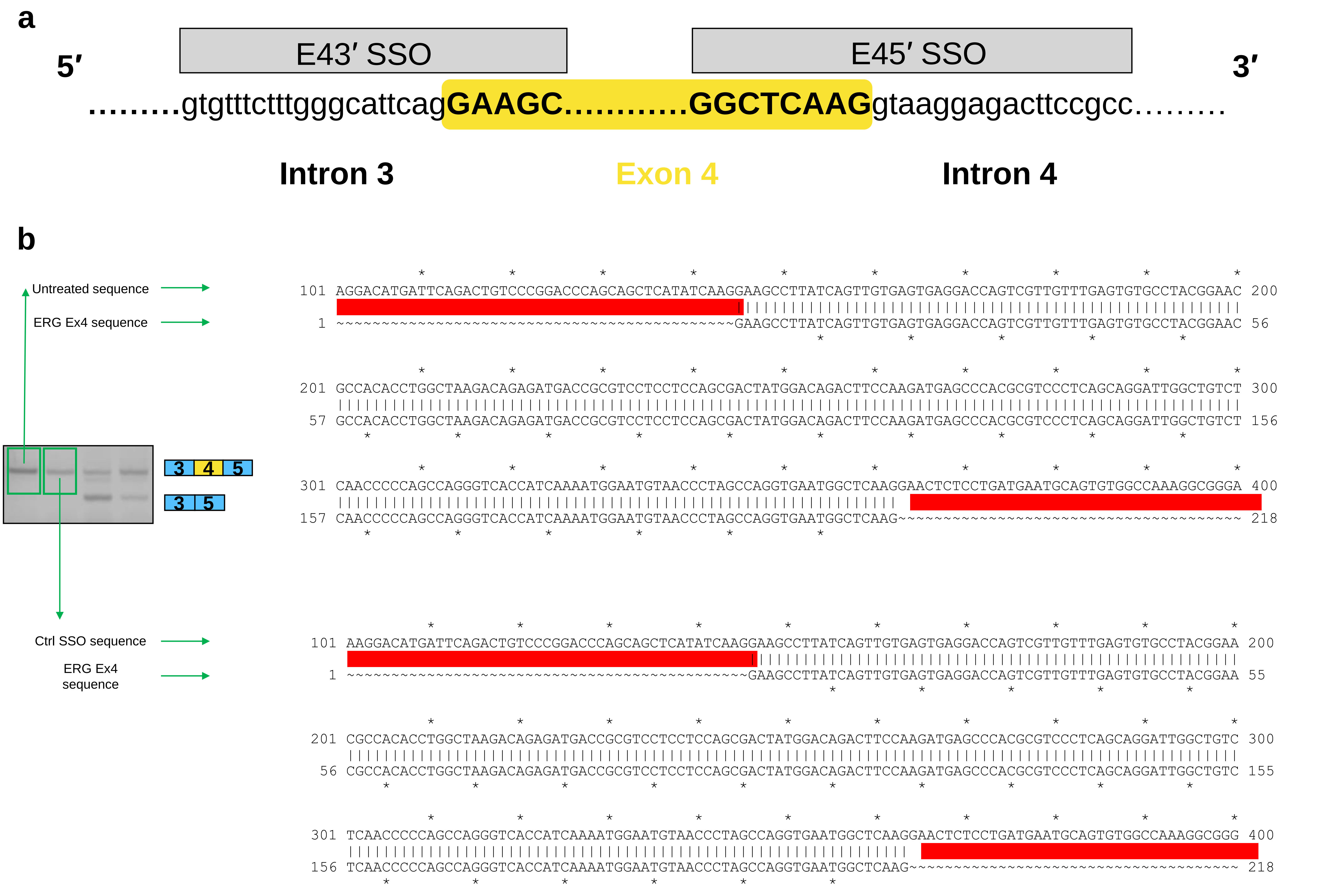
a
E43′ SSO
E45′ SSO
5′
3′
………gtgtttctttgggcattcagGAAGC…………GGCTCAAGgtaaggagacttccgcc………
Intron 3
Exon 4
Intron 4
b
Untreated sequence
ERG Ex4 sequence
3 4 5
3 5
Ctrl SSO sequence
ERG Ex4 sequence
101 AGGACATGATTCAGACTGTCCCGGACCCAGCAGCTCATATCAAGGAAGCCTTATCAGTTGTGAGTGAGGACCAGTCGTTGTTTGAGTGTGCCTACGGAAC 200
1 ~~~~~~GAAGCCTTATCAGTTGTGAGTGAGGACCAGTCGTTGTTTGAGTGTGCCTACGGAAC 56
201 GCCACACCTGGCTAAGACAGAGATGACCGCGTCCTCCTCCAGCGACTATGGACAGACTTCCAAGATGAGCCCACGCGTCCCTCAGCAGGATTGGCTGTCT 300
57 GCCACACCTGGCTAAGACAGAGATGACCGCGTCCTCCTCCAGCGACTATGGACAGACTTCCAAGATGAGCCCACGCGTCCCTCAGCAGGATTGGCTGTCT 156
301 CAACCCCCAGCCAGGGTCACCATCAAAATGGAATGTAACCCTAGCCAGGTGAATGGCTCAAGGAACTCTCCTGATGAATGCAGTGTGGCCAAAGGCGGGA 400
157 CAACCCCCAGCCAGGGTCACCATCAAAATGGAATGTAACCCTAGCCAGGTGAATGGCTCAAG~~~~~~~~~~~~~~~~~~~~~~~~~~~~~~~~~~~~~ 218
101 AAGGACATGATTCAGACTGTCCCGGACCCAGCAGCTCATATCAAGGAAGCCTTATCAGTTGTGAGTGAGGACCAGTCGTTGTTTGAGTGTGCCTACGGAA 200
1 ~~~~~~GAAGCCTTATCAGTTGTGAGTGAGGACCAGTCGTTGTTTGAGTGTGCCTACGGAA 55
201 CGCCACACCTGGCTAAGACAGAGATGACCGCGTCCTCCTCCAGCGACTATGGACAGACTTCCAAGATGAGCCCACGCGTCCCTCAGCAGGATTGGCTGTC 300
56 CGCCACACCTGGCTAAGACAGAGATGACCGCGTCCTCCTCCAGCGACTATGGACAGACTTCCAAGATGAGCCCACGCGTCCCTCAGCAGGATTGGCTGTC 155
301 TCAACCCCCAGCCAGGGTCACCATCAAAATGGAATGTAACCCTAGCCAGGTGAATGGCTCAAGGAACTCTCCTGATGAATGCAGTGTGGCCAAAGGCGGG 400
156 TCAACCCCCAGCCAGGGTCACCATCAAAATGGAATGTAACCCTAGCCAGGTGAATGGCTCAAG~~~~~~~~~~~~~~~~~~~~~~~~~~~~~~~~~~~~~ 218

Supp Fig.1

b continued

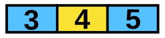

E43′ SSO full length sequence

ERG Ex4 sequence

```
             *         *         *         *         *         *         *         *         *         *
101 GAAGGACATGATTCAGACTGTCCCGGACCCAGCAGCTCATATCAAGGAAGCCTTATCAGTTGTGAGTGAGGACCAGTCGTTGTTTGAGTGTGCCTACGGA 200
                                                  ||||||||||||||||||||||||||||||||||||||||||||||||||||||
  1 ~~~~~~~~~~~~~~~~~~~~~~~~~~~~~~~~~~~~~~~~~~~~~~GAAGCCTTATCAGTTGTGAGTGAGGACCAGTCGTTGTTTGAGTGTGCCTACGGA 54
                                                           *         *         *         *         *

             *         *         *         *         *         *         *         *         *         *
201 ACGCCACACCTGGCTAAGACAGAGATGACCGCGTCCTCCTCCAGCGACTATGGACAGACTTCCAAGATGAGCCCACGCGTCCCTCAGCAGGATTGGCTGT 300
    ||||||||||||||||||||||||||||||||||||||||||||||||||||||||||||||||||||||||||||||||||||||||||||||||||||
 55 ACGCCACACCTGGCTAAGACAGAGATGACCGCGTCCTCCTCCAGCGACTATGGACAGACTTCCAAGATGAGCCCACGCGTCCCTCAGCAGGATTGGCTGT 154
         *         *         *         *         *         *         *         *         *         *

             *         *         *         *         *         *         *         *         *         *
301 CTCAACCCCCAGCCAGGGTCACCATCAAAATGGAATGTAACCCTAGCCAGGTGAATGGCTCAAGGAACTCTCCTGATGAATGCAGTGTGGCCAAAGGCGG 400
    ||||||||||||||||||||||||||||||||||||||||||||||||||||||||||||||||
155 CTCAACCCCCAGCCAGGGTCACCATCAAAATGGAATGTAACCCTAGCCAGGTGAATGGCTCAAG~~~~~~~~~~~~~~~~~~~~~~~~~~~~~~~~~~~~ 218
         *         *         *         *         *         *
```

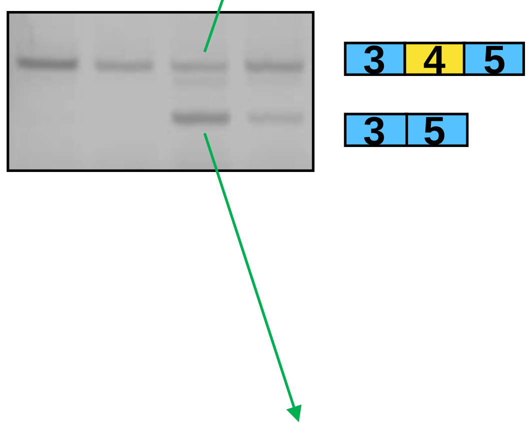

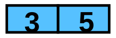

E43′ SSO skipped sequence

ERG Ex4 sequence

```
             *         *         *         *         *         *         *         *         *         *
101 GACATGATTCAGACTGTCCCGGACCCAGCAGCTCATATCAAGGAACTCTCCTGATGAATGCAGTGTGGCCAAAGGCGGGAAGATGGTGGGCAGCCCAGAC 200
    ....................................................................................................
  1 ~~~~~~~~~~~~~~~~~~~~~~~~~~~~~~~~~~~~~~~~~~~~~~~~~~~~~~~~~~~~~~~~~~~~~~~~~~~~~~~~~~~~~~~~~~~~~~~~~~~~ 1

             *         *         *         *         *         *         *         *         *         *
201 ACCGTTGGGATGAACTACGGCAGCTACATGGAGGAGAAGCACATGCCACCCCCAAACATGACCACGAACGAGCGCAGAGTTATCGTGCCAGCAGATCCTA 300
    ....................................................................................................
  1 ~~~~~~~~~~~~~~~~~~~~~~~~~~~~~~~~~~~~~~~~~~~~~~~~~~~~~~~~~~~~~~~~~~~~~~~~~~~~~~~~~~~~~~~~~~~~~~~~~~~~ 1

             *         *         *         *         *         *         *         *         *         *
301 CGCTATGGAGTACAGACCATGTGCGGCAGTGGCTGGAGTGGGCGGTGAAAGAATATGGCCTTCCAGACGTCAACATCTTGTTATTCCAGAACATCGATGG 400
    ....................................................................................................
  1 ~~~~~~~~~~~~~~~~~~~~~~~~~~~~~~~~~~~~~~~~~~~~~~~~~~~~~~~~~~~~~~~~~~~~~~~~~~~~~~~~~~~~~~~~~~~~~~~~~~~~ 1
```

Supp Fig.1 continued

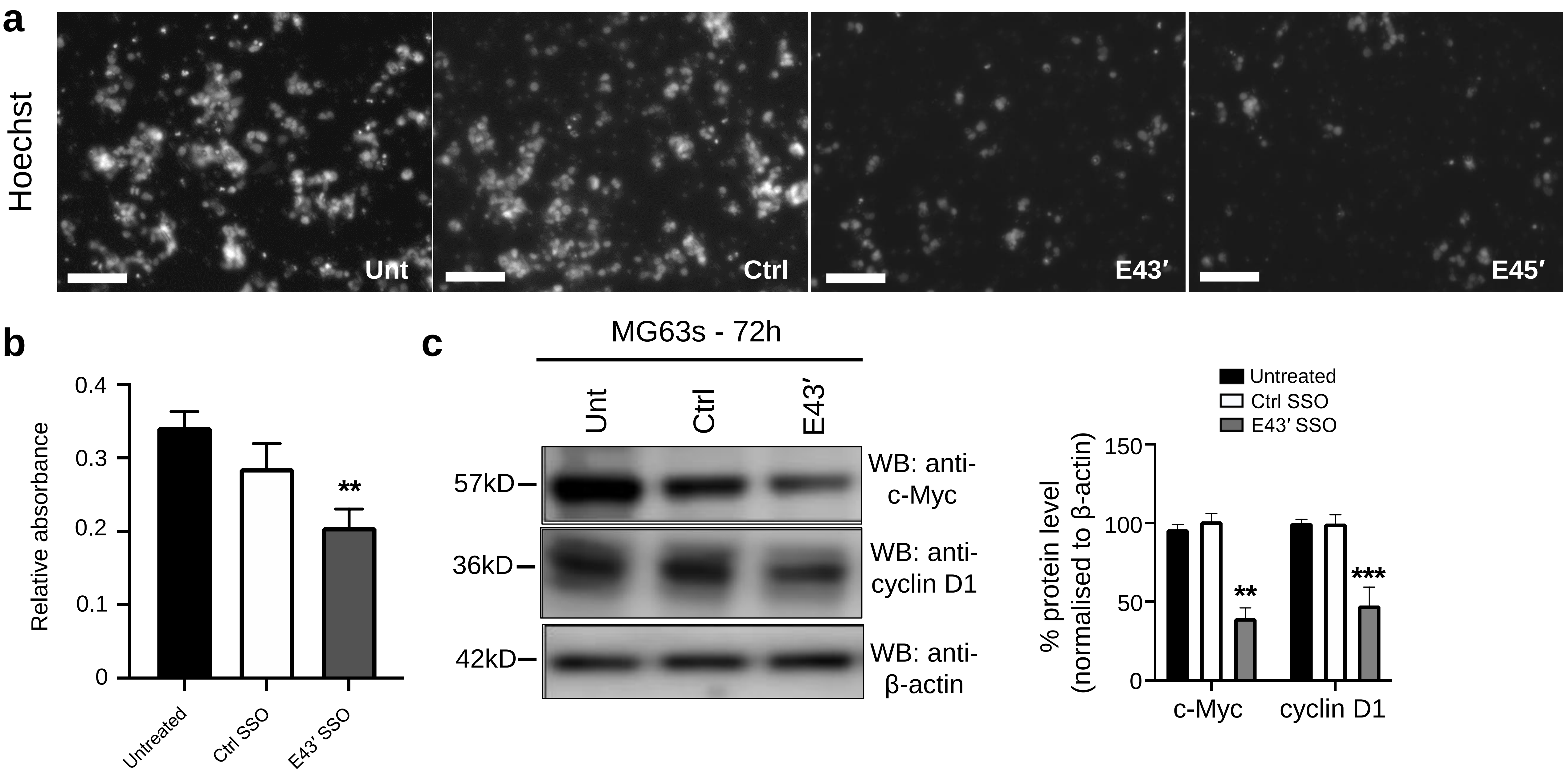
a
Hoechst
Unt
Ctrl
E43′
E45′
b
Relative absorbance
0.4
0.3
0.2
0.1
0
**
Untreated
Ctrl SSO
E43′ SSO
c
MG63s - 72h
Unt
Ctrl
E43′
57kD
36kD
42kD
WB: anti-c-Myc
WB: anti-cyclin D1
WB: anti-β-actin
Untreated
Ctrl SSO
E43′ SSO
% protein level (normalised to β-actin)
150
100
50
0
**
***
c-Myc
cyclin D1

Supp Fig.2

a

E43' SSO: 5'-GCTTCCTGAATGCCCAAAGAAACAC-3'

E45' SSO: 5'-GGCGGAAGTCTCCTTACCTTGAGCC-3'

RED: EXON 4

BLUE: SSOs

INTRON SEQUENCE: BLACK, ITALICS

```
                                                            E43'SSO
                                                     GTGTTTCTTTGGGCATTCA
Human       ----------------------TTGGAGTGTCTTGAATTATGTGTTTCTTTGGGCATTCA   38
Mouse       TCAGCACCACGTTGCTGTCCTCTGGGATGTCCTGAGTCTGCGTTTCTGTTTCTTCGTCCA   60
                                  * ***    **     *  ** * * ***   * * **

            GGAAGC
Human       GGAAGCCTTATCAGTTGTGAGTGAGGACCAGTCGTTGTTTGAGTGTGCCTACGGAACGCC   98
Mouse       GGAGGCCTTGTCAGTTGTGAGCGAGGACCAGTCACTATTTGAGTGTGCCTACGGAACGCC   120
            *** ***** *********** ***********  * ***********************

Human       ACACCTGGCTAAGACAGAGATGACCGCGTCCTCCTCCAGCGACTATGGACAGACTTCCAA   158
Mouse       ACACCTGGCTAAGACAGAGATGACCGCATCCTCTTCCAGTGACTATGGCCAGACATCCAA   180
            *************************** ***** ***** ******** ***** *****

Human       GATGAGCCCACGCGTCCCTCAGCAGGATTGGCTGTCTCAACCCCCAGCCAGGGTCACCAT   218
Mouse       GATGAGTCCCAGAGTCCCTCAGCAGGACTGGCTGTCTCAAGCCCCAGCCAGGGTCACCAT   240
            ****** **  * ************** ************ *******************

                                                   E45'SSO
                                           GGCTCAAGGTAAGGAGACTTCCGCC
Human       CAAAATGGAATGTAACCCTAGCCAGGTGAATGGCTCAAGGTAAGGAGACTTCCGCCCCTT   278
Mouse       CAAGATGGAGTGCAACCCTAGTCAGGTGAATGGTTCCAGGTAAGCAATGCTGACCCTGCC   300
            *** ***** ** ******** *********** ** ******* *    *   **

Human       TCTCTCCCTGACCTCCCATTTT--------------     300
Mouse       TGTCCTGG--ACCTCTATTCTTCCCATCGGTAGCTT     334
            * **      *****   * **
```

b

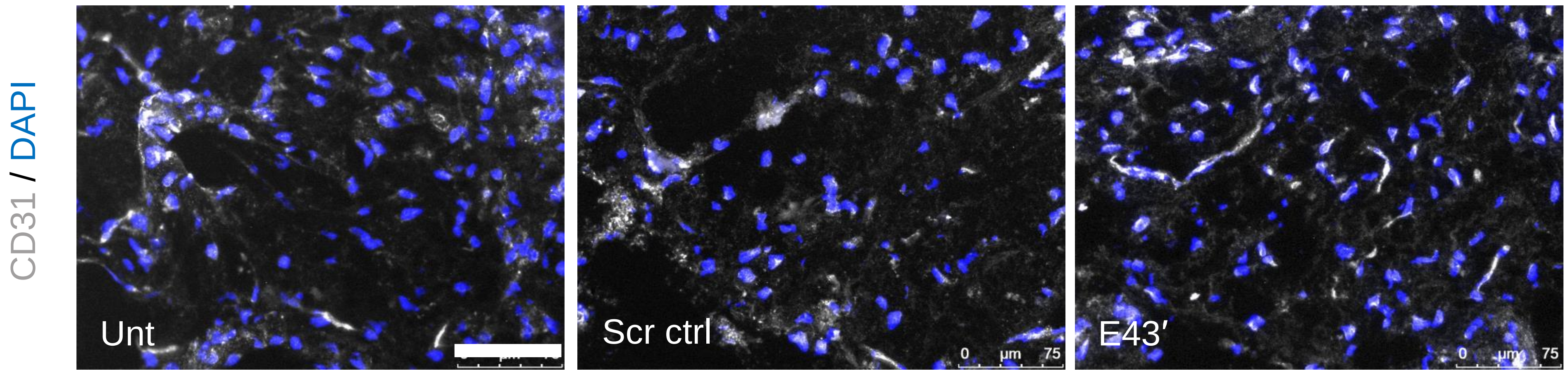

c

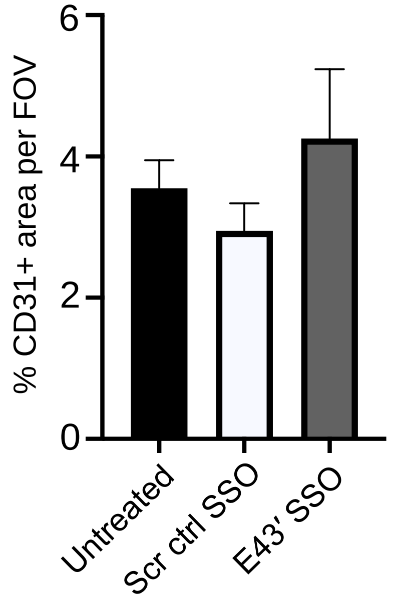

Supp Fig.3

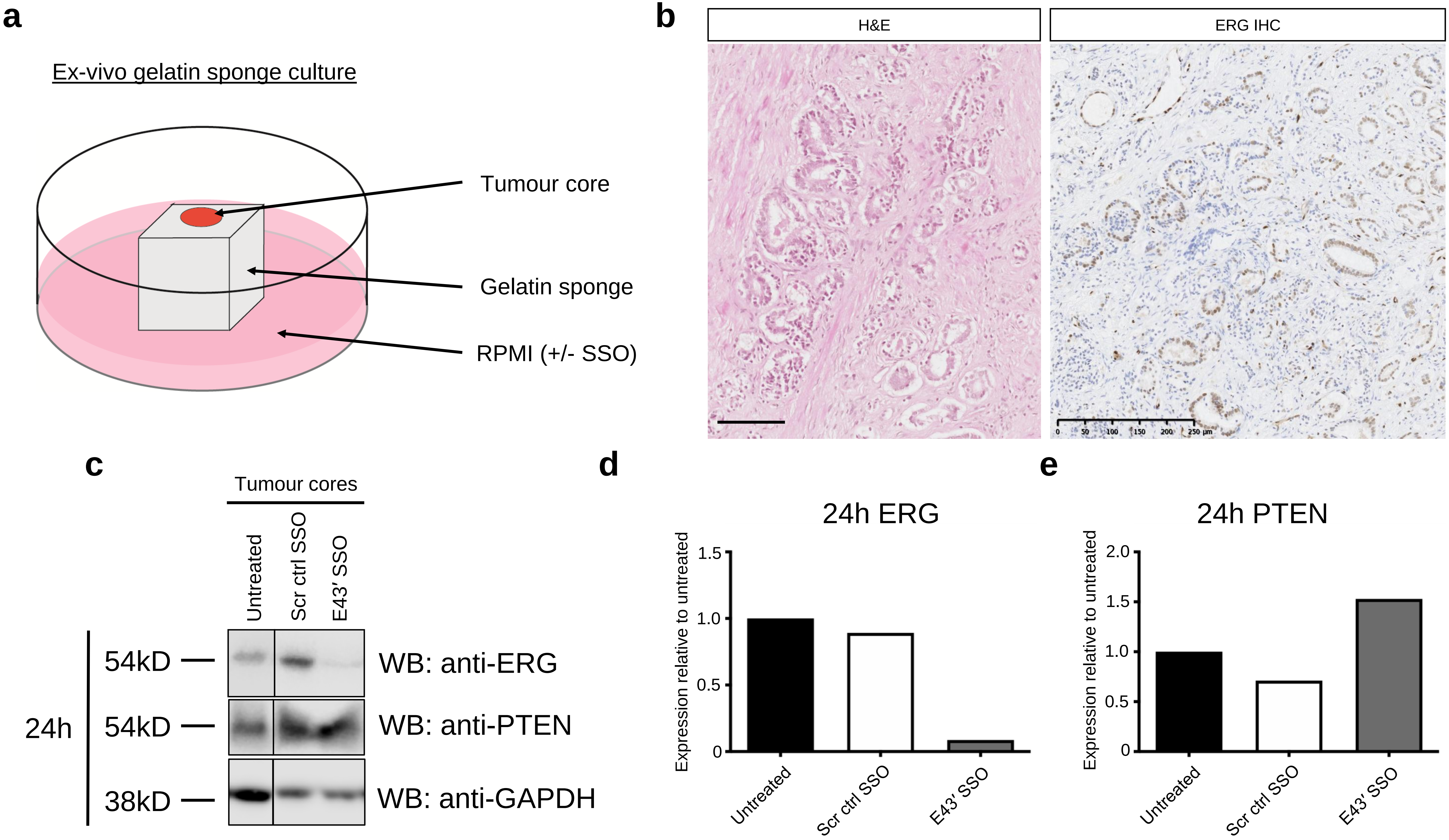

a
Ex-vivo gelatin sponge culture
Tumour core
Gelatin sponge
RPMI (+/- SSO)
b
H&E
ERG IHC
c
Tumour cores
Untreated
Scr ctrl SSO
E43' SSO
24h
54kD
54kD
38kD
WB: anti-ERG
WB: anti-PTEN
WB: anti-GAPDH
d
24h ERG
Expression relative to untreated
Untreated
Scr ctrl SSO
E43' SSO
e
24h PTEN
Expression relative to untreated
Untreated
Scr ctrl SSO
E43' SSO

Supp Fig.4
